# Accuracy of Neck Circumference in Classifying Overweight and Obese US Children

**DOI:** 10.1155/2014/781841

**Published:** 2014-01-30

**Authors:** Youngwon Kim, Jung-Min Lee, Kelly Laurson, Yang Bai, Glenn A. Gaesser, Gregory J. Welk

**Affiliations:** ^1^Department of Kinesiology, Iowa State University, 235 Forker Building, Ames, IA 50011, USA; ^2^School of Kinesiology and Recreation, Illinois State University, Campus Box 5120, 250 McCormick Hall, Normal, I1 61790, USA; ^3^School of Nutrition and Health Promotion, Arizona State University, 502 East Monroe Street, Suite C 250, Phoenix, AZ 85004, USA

## Abstract

*Objective.* To evaluate classification accuracy of NC and compare it with body mass index (BMI) in identifying overweight/obese US children. *Methods.* Data were collected from 92 children (boys: 61) aged 7 to 13 over a 2-year period. NC, BMI, and percent of body fat (BF%) were measured in each child and their corresponding cut-off values were applied to classify the children as being overweight/obese. Classification accuracy of NC and BMI was systematically investigated for boys and girls in relation to true overweight/obesity categorization as assessed with a criterion measure of BF% (i.e., Bod Pod). *Results.* For boys, Cohen's *κ* (0.25), sensitivity (38.1%), and specificity (85.0%) of NC were smaller in comparison with Cohen's *κ* (0.57), sensitivity (57.1%), and specificity (95.0%) of BMI in relation to BF% categorization. For girls, Cohen's *κ* (0.45), sensitivity (50.0%), and specificity (91.3%) of NC were smaller in comparison with Cohen's *κ* (0.52), sensitivity (50.0%), and specificity (95.7%) of BMI. *Conclusion.* NC measurement was not better than BMI in classifying childhood overweight/obesity and, for boys, NC was inferior to BMI. Pediatricians and/or pediatric researchers should be cautious or wary about incorporating NC measurements in their pediatric care and/or research.

## 1. Introduction

The prevalence of childhood overweight/obesity in the US has increased during the past 30 years [1]. Childhood overweight/obesity is associated with health risk factors both during childhood [2, 3] and adulthood [4, 5], and with tracking to adulthood obesity [6–8]. Consequently, identification of overweight/obese children early in life may be an important part of an overall health screening process that could be used to improve well-being in this population [9, 10].

The most commonly used screening tool for detecting childhood overweight/obesity is the body mass index (BMI; weight (kg)/height (m) squared). The standard method used in the United States relies on the use of gender and age-specific BMI growth charts from the Centers for Disease Control and Prevention (CDC) [11]. Youth above the standard 85th percentile are considered overweight while youth above the 95th percentile are considered obese.

While the BMI is widely used and accepted, there has been recent interest in the use of neck circumference (NC) as an alternative screening method. A study by Nafiu et al. [12] established age- and gender-specific cut-offs for NC using receiver operating characteristics curve (ROC curve) on a large sample of children (*n* = 1102, 52% boys, aged 6 to 18 yrs). The analyses were designed to maximize both sensitivity and specificity of NC cut-offs in relation to the overweight/obesity categorization using the CDC growth charts for BMI (i.e., values above the 85th percentile) [11]. This methodology resulted in a set of age- and gender-specific NC cut-offs that ranged from 28.5 cm to 39.0 cm for boys and from 27.0 cm to 34.6 cm for girls [12].

A limitation of the previous study is that it only linked NC values to BMI rather than to a more appropriate criterion. Before the NC values can be used in clinical practice, it is important to systematically validate the published NC cut-off values in separate studies prior to its widespread use in clinical settings. To date, however, the Nafiu et al.'s NC cut-offs [12] for classifying overweight/obese children have not been evaluated using an appropriate gold standard or in an independent sample of children. Therefore, the purpose of this study was to compare the relative accuracy of the Nafiu et al.'s NC cut-offs [12] in the classification of overweight/obesity against the BMI percentiles [11] using estimates of percent of body fat (BF%) from whole body air-displacement plethysmography as a criterion measure of body composition.

## 2. Materials and Method

### 2.1. Study Design

This study was conducted as an ancillary study of a 5-year National Institute of Health-funded research Project (R01 HL910006) designed to validate varying types of accelerometer-based physical activity monitors in children. The procedures and protocol of the study were reviewed and approved by the local Institutional Review Board. Written parental consent and children's assent were obtained after informing them about the procedures and purpose of the study and prior to the participation. The data for the present study were collected in the summer of 2011 and 2012. A total of 92 children had data on the needed measures (2011: *n* = 60; 2012: *n* = 32). In Table 1, the characteristics of the included participants are summarized.

### 2.2. Anthropometric Measurement

Neck circumference (NC) was measured using a flexible ruler tape on the mid-point of the neck at the level of the thyroid cartilage, with a participant's body held erect, eyes facing forward, and normal breathing [12]. Standing height and body weight were measured using a wall-mounted stadiometer (*Harpenden, London, UK*) and electronic scale (*2008 Sunbeam products, Inc., Boca Raton, FL*) to the nearest 0.1 cm and 0.1 kg, respectively. BMI was calculated by dividing weight in kilograms by the square of height in meters.

BF% was measured via whole body air-displacement plethysmography with the Bod Pod (*Life Measurement, Inc., Concord, CA*), which has been considered a valid and reliable method for BF% measurement [13, 14]. The Bod Pod was calibrated according to the manufacturer's guidelines prior to each testing. Participants were wearing a tight fitting swimsuit and swim cap and were asked to sit still and not to talk in the chamber during the measurement. Body volume measurements were carried out twice and the average value of the two measurements was taken into consideration for analyses. BF% was estimated using the Lohman et al. equation [15, 16] programmed in the Bod Pod.

### 2.3. Data Processing/Statistical Analyses

The Nafiu et al.'s age- and gender-specific NC cut-offs [12] and the CDC growth charts for BMI [11] were applied to categorize NC and BMI values, respectively, as normal weight or overweight/obese. A set of criterion referenced health standards for BF% were used to determine the “true” classification of overweight/obesity for the participants [17, 18]. These standards (established using nationally representative data from the National Health and Nutrition Examination Survey) were found to have a sensitivity of 95.0% and specificity of 71.0% for boys and a sensitivity of 96.8% and specificity of 68.9% for girls for predicting risk of metabolic syndrome in a similarly aged population [17, 18]. Thus, they provide a defensible standard to evaluate the accuracy of these alternative screening tools available for body composition screening. The cut-off values used for NC, BMI, and BF% calculations are provided in Table 2.

All the statistical analyses were conducted using STATA/SE Version 10.0 for Windows (StataCorp LP, College Station, TX). Mean (*M*), standard deviations (SD), and minimum and maximum values of the anthropometric variables were calculated. Pearson correlation coefficients along with corresponding two-sided *P* values were obtained among NC, BMI, and BF%. A level of significance was set at 0.05. Classification accuracy of the Nafiu et al.'s NC cut-offs [12] and the CDC growth charts for BMI [11] in identifying overweight/obese children were evaluated against the standard cut-offs for BF% [17, 18] using weighted Cohen's kappa coefficient (Cohen's *κ*), sensitivity, specificity, positive predictive value, and negative predictive value. Cohen's *κ* is an index used to evaluate classification agreement between two screening tools for categorical variables, with agreement by chance corrected. Cohen's *κ* is categorized as “no agreement” (*κ* ≤ 0), “slight” (0 < *κ* ≤ 0.20), “fair” (0.20 < *κ* ≤ 0.40), “moderate” (0.40 < *κ* ≤ 0.60), “substantial” (0.60 < *κ* ≤ 0.80), and “almost perfect” agreement (0.80 < *κ* ≤ 1.00) [19]. Sensitivity refers to the ability of a certain screening method (i.e., NC or BMI in the context of the present study) to precisely detect a disease (i.e., overweight/obese) in people who indeed have the disease (i.e., as assessed with BF%), while specificity is referred to as the ability of a screening method to identify the absence of a disease (i.e., normal weight) in a population without the disease. Positive predictive value represents the proportion of people who have a disease (i.e., overweight/obese) among people who test positive. Negative predictive value is the proportion of people without a disease (i.e., normal weight) among people who test negative.

## 3. Results

The Nafiu et al.'s NC cut-offs [12] classified 13 (21.3%) of 61 boys and 6 (19.4%) of 31 girls as being overweight/obese. The CDC growth charts for BMI [11] classified 14 boys (23.0%) and 5 girls (16.1%) as being overweight/obese. Twenty one boys (34.4%) and 8 girls (25.8%) were overweight/obese in accordance with the standard cut-offs for BF% [17, 18].

Table 3 shows statistically significant positive correlations among the three different measures: NC, BMI, and BF%. For boys, the correlation between NC and BF% values (*r* = 0.44, *P* < 0.001) was smaller in comparison to the correlation between BMI and BF% (*r* = 0.76, *P* < 0.001) and between NC and BMI (*r* = 0.78, *P* < 0.001). For girls, a similar pattern was observed that the correlation between NC and BF% (*r* = 0.65, *P* < 0.001) was smaller than the correlation between BMI and BF% (*r* = 0.74, *P* < 0.001) and between NC and BMI (*r* = 0.72, *P* < 0.001).

Table 4 summarized Cohen's *κ*, sensitivity, specificity, positive predictive value, and negative predictive value for both the Nafiu et al.'s NC cut-offs [12] and the CDC growth charts for BMI [11] in relation to the standard cut-offs for BF% [17, 18] for boys and girls. For boys, the Nafiu et al.'s NC cut-offs [12] showed “fair” classification agreement (Cohen's *κ*; 0.25) with the standard cut-offs for BF% [17, 18] while “moderate” classification agreement (Cohen's *κ*; 0.57) was observed between the CDC growth charts for BMI [11] and the standard cut-offs for BF% [17, 18]. Sensitivity (38.1%), specificity (85.0%), positive predictive value (57.1%), and negative predictive value (72.3%) of the Nafiu et al.'s NC cut-offs [12] were smaller in comparison with sensitivity (57.1%), specificity (95.0%), positive predictive value (85.7%), and negative predictive value (80.9%) of the CDC growth charts for BMI [11]. For girls, the Nafiu et al.'s NC cut-offs [12] demonstrated “moderate” classification agreement (Cohen's *κ*; 0.45) but was smaller than the CDC growth charts for BMI [11] (Cohen's *κ*; 0.52) in relation to the standard cut-offs for BF% [17, 18]. Sensitivity (50.0%), specificity (91.3%), positive predictive value (66.7%), and negative predictive value (84.0%) of the Nafiu et al.'s NC cut-offs [12] were smaller (or equivalent) in comparison with sensitivity (50.0%), specificity (95.7%), positive predictive value (80.0%), and negative predictive value (84.6%) of the CDC growth charts for BMI [11] relative to the standard cut-offs for BF% [17, 18].

## 4. Discussion

In the present study, classification accuracy of the recently developed set of Nafiu et al.'s NC cut-offs [12] for identifying overweight/obese children was examined in relation to the gold standard (i.e., Bod Pod) for body composition measurement. Overall, the Nafiu et al.'s NC cut-offs [12] demonstrated good classification accuracy of overweight/obesity for girls, but low for boys. However, they [12] did not prove superiority over the traditional overweight/obesity classification method, BMI assessed with the CDC growth charts [11].

Such limited classification accuracy of the Nafiu et al.'s NC cut-offs [12] appears to be due to the following two reasons. First, the CDC growth charts for BMI [11] may not be accurate enough to serve as a reference method for developing a precise set of NC cut-offs. While BMI has been considered a useful screening tool for epidemiological studies with large sample sizes [11], it tends to yield biased estimates of total fat distributions at an individual level [20], thereby limiting the practice of BMI as a “gold standard” measure in identifying overweight/obese children. This may have impaired the accuracy of the NC cut-offs developed in the Nafiu et al.'s calibration study [12]. The second potential reason is the inclusion of a large number of children (i.e., 70% of the total) in the Nafiu et al.'s calibration study [12] that were undergoing various types of outpatient surgeries at a children's hospital. Thus, the NC cut-offs [12] developed for this particular group of children appeared not to precisely classify overweight/obesity when applied for the healthy randomly selected children included in the present study. For the purpose of verifying this issue, we performed subsequent analyses (i.e., unreported herein) to obtain sensitivity and specificity of Nafiu et al.'s NC cut-offs [12] relative to BMI as a reference method and then to compare them with the sensitivity and specificity that were reported in the original calibration study of Nafiu et al. [12]. The present study showed lower sensitivity (i.e., 38.1% for boys and 50.0% for girls) and greater specificity (i.e., 85.0% for boys and 91.3% for girls) for Nafiu et al.'s NC cut-offs [12] in comparison with the sensitivity (i.e., 82.5% for boys and 79.7% for girls) and specificity (i.e., 83.7% for boys and 82.8% for girls) reported in the subjects studied by Nafiu et al. [12]. These differential results between the present study and that of Nafiu et al. [12] may be indicative of lower classification accuracy of NC cut-offs [12] when used in nonpatient populations.

In addition to the set of NC cut-offs by Nafiu et al. [12], other sets of NC cut-offs were independently established in an effort to identify overweight/obesity for other populations such as adults [21] and Turkish children [22]. These two studies [21, 22] also used BMI as a reference method in developing the NC cut-offs from ROC-curve analyses, thereby potentially limiting the classification accuracy of the developed NC cut-offs. Also, as discussed above, the usual practice of applying the NC cut-offs developed from patients [21] to nonpatient populations may be unwarranted. As such, the classification accuracy of NC cut-offs for patients may be maximized when applied only for specific patient populations. Moreover, all of the above-mentioned studies [12, 21, 22] advocated the use of NC measurement, primarily based on its “practicality” for clinical settings. Some of the practical features of NC measurement discussed in the studies [12, 21, 22] were as follows: easy/simple/inexpensive to use, unnecessary to remove upper clothes, and less susceptible to harsh weather than other measures (i.e., waist circumference measure). The results of the present study, however, suggest that the accuracy of NC measurement is not so reasonably high that pediatricians/pediatric educators may not be able to capitalize on the good “practicality” per se.

NC measurement may not be precise enough to serve as a stand-alone alternative to BMI. In support, in the present study, BMI showed better (or comparable) classification accuracy of overweight/obesity for boys and girls, respectively, in comparison with NC. Moreover, BMI has been recommended by numerous previous studies [9–11, 23] as a useful screening tool to identify childhood overweight/obesity. Therefore, it has become a tradition in nearly all pediatric clinical settings that height and weight are routinely measured as part of their basic check-ups for a child to yield his or her corresponding BMI value. For these reasons, unless classification accuracy of NC measurement for childhood overweight/obesity is strongly supported with scientific evidence, NC measurement may not be broadly used in clinical practice, despite its high practicality.

To date, a relatively large number of studies have been conducted to examine the associations between NC and varying health indicators in children (i.e., cardiovascular risk factors [24–26], prehypertension [27], and perioperative adverse respiratory events [28]). However, a limited body of scientific evidence has been established to determine whether or not NC measurement can serve as a useful tool for classifying childhood overweight/obesity. To our knowledge, this is the first study examining the efficacy of NC measurement in classifying childhood overweight/obesity in relation to a criterion measure of body composition in an independent sample of US children. In order for NC measurement to be widely adopted in clinical practice, therefore, additional studies are needed (1) to develop and/or (2) to evaluate a set of NC cut-offs relative to a gold-standard reference (i.e., Bod Pod, dual-energy X-ray absorptiometry) for body composition measurement with average populations of children (rather than with individuals undergoing surgeries).

Some limitations need to be taken into consideration when interpreting the results of the present study. First of all, a relatively small number of children participated in the study. Therefore, it was challenging to include a sufficient number of children in each age (i.e., 7–13 yrs), which limited the analysis and stratification of the results by age. However, since the statistical methods (e.g., Cohen's *κ*, sensitivity, specificity, positive predictive value, and negative predictive value) used herein are not biased with a small sample, the application of the (age-specific) NC [12], BMI [11], and %BF [17, 18] cut-offs for each age yielded unbiased estimates of classification accuracy. Another limitation was the inability to compare the accuracy of NC with other types of anthropometric measures (i.e., waist circumference, waist-to-hip ratio). However, NC was directly compared with the most commonly used classification method, BMI.

## 5. Conclusions

The results of this study appear not to strongly support the use of NC measurement as a useful screening tool for classifying childhood overweight/obesity. While NC measurement holds great practicality, its unsatisfactory accuracy in overweight/obesity classification may preclude the widespread use at clinical settings. Pediatricians and/or pediatric researchers should be informed of the accuracy of NC measurement in childhood overweight/obesity classification prior to incorporating it in their practical pediatric care and/or research.

## Figures and Tables

**Table 1:** Descriptive statistics of the included participants (*n* = 92).

Anthropometric measures	Boys (*n* = 61)		Girls (*n* = 31)	
	M	SD	M	SD
Age (yrs)	9.8	2.0	9.6	1.8
Height (cm)	146.1	14.6	140.6	10.1
Weight (kg)	40.2	15.5	34.6	8.3
Neck Circumference (cm)	30.0	3.1	28.7	2.2
Body Mass Index (BMI)	18.3	4.0	17.3	2.5
Percent body fat (%)	18.5	7.9	18.4	6.1

**Table 2:** Cut-off values of neck circumference (NC), body mass index (BMI), and percent body fat (BF%) for classifying overweight/obese children.

Age (yrs)	NC (cm) by Nafiu et al. [12]		BMI^a^		BF%^b^	
	Boys	Girls	Boys	Girls	Boys	Girls
7	28.7	27.1	17.4	17.6	17.5	19.5
8	29.0	27.9	18.0	18.3	18.9	20.9
9	30.5	29.3	18.6	19.1	20.7	22.7
10	32.0	30.5	19.4	20.0	22.5	24.4
11	32.2	31.0	20.2	20.9	23.7	25.8
12	32.5	31.1	21.0	21.7	23.7	26.8
13	33.5	31.3	21.9	22.6	22.9	27.8

^a^Centers for Disease Control and Prevention growth charts (5th–85th percentile being normal weight, ≥85th percentile being overweight/obese for boys and girls).

^b^Percent body fat standards (<69th percentile being normal weight for boys, ≥69th percentile being overweight/obese for boys, <68th percentile being normal weight for girls, ≥69th percentile being overweight/obese for girls).

**Table 3:** Pearson correlation coefficients among neck circumference (NC), body mass index (BMI), and percent body fat (BF%) (*n* = 92).

	NC and BF%	BMI and BF%	NC and BMI
Boys (*n* = 61)	.44	.76	.78
Girls (*n* = 31)	.65	.74	.72

Note: all correlations were significant (*P* < 0.001).

**Table 4:** Comparisons of neck circumference (NC), body mass index (BMI), and percent body fat (BF%) in classification of overweight/obese children (*n* = 92).

	NC by Nafiu et al. [12] versus BF%		BMI versus BF%	
	Point estimate	95% CI	Point estimate	95% CI
Boys (*n* = 61)				
Weighted Cohen's kappa (*κ*)	.25	0, .48	.57	.30, .74
Sensitivity (%)	38.1	25.9, 50.3	57.1	44.7, 69.6
Specificity (%)	85.0	76.4, 94.0	95.0	89.5, 100
Positive predictive value (%)	57.1	44.7, 69.6	85.7	76.9, 94.5
Negative predictive value (%)	72.3	61.1, 83.6	80.9	71.0, 90.7
Girls (*n* = 31)				
Weighted Cohen's kappa (*κ*)	.45	.08, .73	.52	.13, .78
Sensitivity (%)	50.0	32.4, 67.6	50.0	32.4, 67.6
Specificity (%)	91.3	81.4, 100	95.7	88.5, 100
Positive predictive value (%)	66.7	50.1, 83.3	80.0	65.9, 94.1
Negative predictive value (%)	84.0	71.1, 96.9	84.6	71.9, 97.3
